# Association Between Hypertensive Disorders of Pregnancy and Long-term Risk of Dilated Cardiomyopathy: Population based cohort study using multiple linked UK electronic health records databases

**DOI:** 10.1001/jamacardio.2025.0328

**Published:** 2025-05-01

**Authors:** Upasana Tayal, Constantinos Kallis, Georgie M Massen, Nora Rossberg, Emily L Graul, Jenni Quint

**Affiliations:** a National Heart Lung Institute, Imperial College London, London, UK; b School of Public Health, Imperial College London, London, UK; c Royal Brompton & Harefield Hospitals, Guy’s and St. Thomas’ NHS Foundation Trust, London; d The George Institute, London

## Abstract

**Importance:**

The impact of hypertensive disorders of pregnancy on developing dilated cardiomyopathy is unknown.

**Objective:**

Determine whether hypertensive disorders of pregnancy are associated with long-term risk of dilated cardiomyopathy.

**Design:**

Population-based cohort study using linked electronic health records databases; Clinical Practice Research Datalink (CPRD) Pregnancy Register, CPRD Aurum (primary care), Hospital Episode Statistics Admitted Patient Care and Office for National Statistics mortality data.

**Setting:**

England.

**Participants:**

Exposed cohort of 14,083 first time pregnant women with hypertensive disorders of pregnancy (assembled January 1997 to December 2018; followed up to July 2023) and unexposed cohort of 70,415 women with normotensive pregnancies randomly sampled from the Pregnancy Register (5:1 ratio).

**Exposure:**

Hypertensive disorder of pregnancy (pre-eclampsia, gestational hypertension).

**Outcome measures:**

Cox proportional hazards models fitted to estimate hazard ratios (HR) of developing dilated cardiomyopathy.

**Results:**

A first-time pregnancy complicated by a hypertensive disorder of pregnancy, compared with a normotensive first-time pregnancy, was associated with a 93% higher risk of developing dilated cardiomyopathy (HRadj 1.93, 95% CI 1.33-2.81, p=0.001; adjusted for maternal age). Dilated cardiomyopathy developed 5.1 (IQR 0.7-10.6) years post-partum in women with HDP and 10.6 (IQR 4.2-15.8) years in women with normotensive first pregnancies. The association remained significant after adjusting for maternal age, birth year, gestational diabetes, post-pregnancy diabetes, post-pregnancy hypertension, total parity, ethnicity and socio-economic status (HRadj 1.55, 95% CI 1.04-2.31, p=0.031). There was a dose response; the association was stronger in women with pre-eclampsia (HRadj 1.85, 95% CI 1.24-2.76, p=0.002) and severe pre-eclampsia (HRadj 4.29, 95% CI 2.32 to 7.96, p<0.001). Maternal age (HRadj per year of age 1.06, 1.03 to 1.08, p<0.001) and post-partum incident hypertension (HRadj 1.68, 1.16-2.42, p=0.006) were independently associated with the development of DCM.

**Conclusions:**

Women with hypertensive disorders of pregnancy had a greater risk of developing dilated cardiomyopathy. Older maternal age and post-partum hypertension were associated with higher risk of dilated cardiomyopathy after a hypertensive disorder of pregnancy.. These findings support long-term clinical vigilance of women with a history of hypertensive disorders of pregnancy.

## Introduction

Hypertensive disorders of pregnancy (HDP) affect 5-10% of pregnancies worldwide^1^. Increasing evidence suggests that HDP are associated with adverse myocardial remodelling after pregnancy^2,3^ but at present, there is limited understanding on long-term cardiomyopathy risk. There is an increasing burden globally of non-ischaemic heart failure, of which dilated cardiomyopathy (DCM) is a leading cause^4^. Genetic and environmental risk factors modify the development of cardiomyopathy^5–9^, but the impact of female-specific reproductive risk factors is unknown. Therefore, to better characterize cardiomyopathy risk in women after a first pregnancy complicated by HDP, we studied DCM incidence in a large nationwide sample using multiple linked electronic health records (EHR) databases.

## Methods

### Data sources

#### CPRD primary care data, linked datasets and the Pregnancy Register

We used routinely collected healthcare data from the Clinical Practice Research Datalink (CPRD) Aurum national primary care database, containing deidentified patient information from consenting general practices (GPs) across the UK, linked to the Pregnancy Register (all pregnancy episodes in the database; 17 million pregnancies), socio-economic deprivation measures, national mortality data and secondary care records (Supplementary Materials). CPRD Aurum is broadly representative of the UK population in terms of age, sex and ethnicity^10–12^.

### Study Population and Exposures

**Figure 1:**
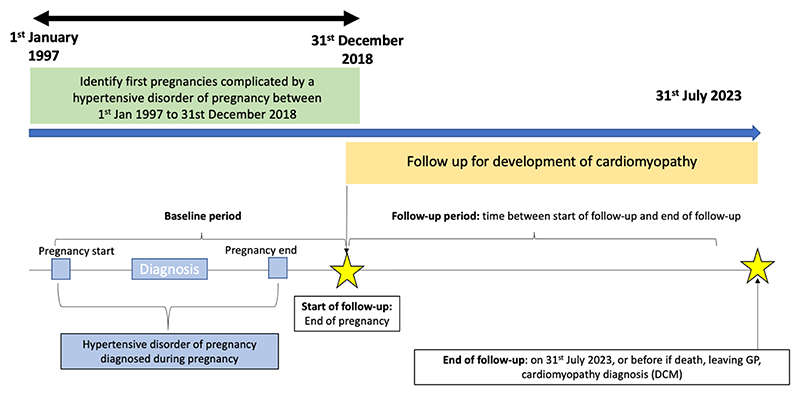
Study overview. Shows timelines for cohort recruitment and follow up.

We included all individuals from the Pregnancy Register with a HDP (pre-eclampsia and gestational hypertension; defined using diagnostic code lists, eTable1, recorded within 12 months of pregnancy start) in a first pregnancy with a known outcome of live birth or stillbirth. A sample with normotensive pregnancies was also randomly sampled from the register in a 5:1 ratio to reduce computational burden. The index pregnancy was included if from 1^st^ Jan 1997 to 31^st^ December 2018 to allow at least 5 years of follow up to 31^st^ July 2023 for the majority of the cohort (Figure 1).

### Outcome

Participants were followed up from the index date (end of pregnancy) until the minimum of the outcome of interest (DCM and non-ischaemic heart failure developing after the end of the pregnancy, defined using codes for primary care and secondary care; eTable 1), leaving the practice, death or practice last collection date.

### Covariates

Maternal age at first pregnancy, birth year, gestational diabetes, postpartumdiabetes, postpartum hypertension, parity, socio-economic status and ethnicity (supplementary materials) were included in analyses.

### Statistical analysis

We fitted Cox proportional hazards models with time between the end of pregnancy to DCM outcome or censoring as duration. Sensitivity analyses (i) restricted to those with a history of pre-eclampsia and severe pre-eclampsia only, and secondary analyses explored additional outcomes (i) of peripartum cardiomyopathy (PPCM) (ii) heart failure and (iii) atherosclerotic cardiovascular disease (ASCVD). All analyses were done using Stata, version 17.

**Ethics and Data Availability statements available in** Supplementary Materials

## Results

The cohort included 14,083 individuals with HDP affecting a first pregnancy (Supplementary Figure 2) and 70,415 individuals with normotensive first pregnancies. Women with a HDP were on average 2 years older than women with unaffected first pregnancies (Table 1). Gestational diabetes, post-pregnancy diabetes and post-pregnancy hypertension were more common in women with a HDP compared with normotensive pregnancies (Table 1). Women with HDP were more likely to be more socio-economically deprived (Table 1).

Median follow up time was 7.6 years (IQR 3.2 - 14.1 years); in the HDP group it was 8.0 years (IQR 3.8 - 13.8 years) vs 7.5 years (3.0-14.1 years) in the group without HDP (p<0.001).

### Development of dilated cardiomyopathy

There were 132 incident cases of DCM, of which 39 were in those with HDP. DCM occurred at a median of 5.1 years (IQR 0.7 to 10.6 years) in the HDP group and 10.6 years (IQR 4.2 to 15.8 years) in the group without HDP, in follow up.

**Figure 2:**
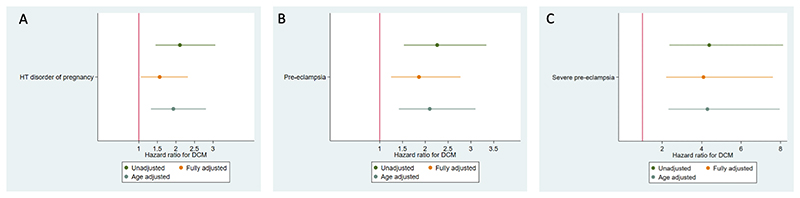
Association between hypertensive disorders of pregnancy and dilated cardiomyopathy. Forest plots of hazard ratio for development of DCM after (Panel A) a first pregnancy complicated by a hypertensive disorder of pregnancy including gestational hypertension, (Panel B) a first pregnancy complicated by pre-eclampsia alone and (Panel C) a first pregnancy complicated by severe pre-eclampsia. Covariates in fully adjusted model = maternal age, birth year, gestational diabetes, post pregnancy diabetes, post pregnancy hypertension, socio-economic status (IMD quintile), ethnicity, final parity. Covariates in age adjusted model = maternal age. DCM = dilated cardiomyopathy.

Compared with women with a normotensive first pregnancy, women with a HDP were at a higher risk of developing DCM (unadjusted HR 2.11, 95% CI 1.45 to 3.06, p<0.001; Figure 2). The association remained significant after adjusting for maternal age (adjusted HR 1.93, 95% CI 1.33 to 2.81, p=0.001) and after additionally adjusting for birth year, gestational diabetes, postpartumdiabetes, postpartum hypertension, parity, race and ethnicity and socio-economic status (HR 1.55, 95% CI 1.04 to 2.31, p=0.031; Figure 1; Supplementary Table 1). The global test for proportional hazards assumption for the final model showed that this assumption holds. Maternal age was independently associated with DCM (adjusted HR per year of age 1.06, 1.03 to 1.08, p<0.001), as was post-partum incident hypertension (adjusted HR 1.68, 1.16-2.42, p=0.006) (Supplementary Table 4).

#### Sensitivity analysis

##### (i) Dose response: Pre-eclampsia

Compared with women with a normotensive first pregnancy, women with pre-eclampsia in their first pregnancy (n=10,918, excluding women with gestational hypertension) were at a higher risk of DCM in adjusted (adjusted HR 1.85, 95% CI 1.24 to 2.76, p=0.002; covariates as per primary analysis) and unadjusted analyses (HR 2.26, 95% CI 1.53 to 3.34, p<0.001; full model Supplementary Table 2). The association was even stronger in women with severe pre-eclampsia (n=1391; adjusted HR 4.09, 95% CI 2.20 to 7.62, p=<0.001; covariates as per primary analysis; unadjusted HR 4.39, 95% CI 2.37 to 8.13, p<0.001) (Figure 1).

#### Secondary analyses

##### (i) PPCM

14 incident cases of PPCM occurred, which were not included in the primary DCM outcome. HDP were strongly associated with PPCM (unadjusted HR 4.86, 95% CI 1.70 to 13.85, p=0.003; full model adjusted HR 4.07, 95% CI 1.33 to 12.49, p=0.014).

##### (ii) HF and ASCVD

Replicating known associations, compared with women with a normotensive first pregnancy, women with a HDP were also at a higher risk of developing heart failure (n=150, adjusted HR 1.87, 95% CI 1.31 to 2.67, p=0.001; adjusted for age) and ASCVD (n=496, adjusted HR 1.48, 95% CI 1.20 to 1.81, p<0.001; adjusted for age) (Supplementary Table 3).

## Discussion

In this nationwide, population-based cohort study, HDP are associated with a twofold greater hazard of DCM. DCM develops on average 5 years after the HDP. Older maternal age at the time of first pregnancy and post-partum incident hypertension were associated with developing DCM in at risk women. This study provides insight into the pathophysiology of DCM and adds to the evidence base to support ongoing surveillance of women with HDP. These findings could represent either a shared biology that predisposes to both HDP and DCM or the potential for HDP to contribute to risk of DCM through a subsequent higher cumulative hypertensive exposure. This study adds to the body of evidence of pleiotropic cardiovascular disease risk following HDP; we replicated associations with ASCVD and heart failure that are consistent in magnitude and temporal onset with other published studies.Earlier cardiomyopathy disease detection is associated with better outcomes. There is a pressing need to understand contributors to cardiomyopathy to identify at risk populations earlier to modify natural history. To date, our understanding of the impact of reproductive risk factors on the development of cardiomyopathy has been limited. These findings may help to inform the timeline of potential follow up and/or surveillance strategies.

At a population level, our study suggests that for every 700 women with HDP, 1 will develop DCM (taking the reciprocal of the absolute risk reduction). Established screening tests for cancer have a number needed to screen from ~500-2500 ^13,14^. Whilst nationally implemented screening programmes require extensive cost-benefit analysis, we propose that patients and physicians be educated as to the potential long-term greater risk of developing DCM after a HDP and be vigilant to symptoms of cardiomyopathy in order to be able to access early appropriate investigation. This should be part of a holistic long term cardiovascular surveillance of women affected by HDP. In parallel, this and other studies support that reproductive history should be an integral part of cardiovascular assessments^15^.

### Limitations

Whilst CPRD data are representative of the UK population, further studies in more racially and ethnically diverse cohorts are needed for broader generalizability. Although a nationwide sample, we were not able to evaluate the risk of recurrent HDPs on DCM due to the limited number of recurrent HDP events. Ascertainment bias may have confounded the results; exposed individuals with HDP may have been more likely to be screened for DCM. However, this is unlikely to be a major limitation, as there is no systematic screening of at-risk patients in the UK. Post partum guidelines focus on antihypertensive management. There may be under-ascertainment as subclinical DCM would not be detected. HDP, PPCM and DCM may have a shared etiology and women with PPCM may be under-represented related to misclassification of PPCM as DCM. Future studies are needed to investigate the potential mechanistic basis of the association between HDP and DCM, including evaluating the genetic basis of these diseases which was not available in this study.

## Conclusion

In this nationwide sample, we find that HDP in a first pregnancy are associated with a two-fold greater hazard of DCM, occurring on average 5 years after the HDP, and 5 years earlier than in women with a normotensive pregnancy. These findings provide insight into the pathophysiology of DCM and may support the long-term clinical monitoring of women after HDP.

## Supplementary Material

Supplementary

## Figures and Tables

**Table 1:** Baseline characteristics in exposed and unexposed groups.

	HDP in first pregnancy n = 14,083	Normotensive first pregnancy n = 70,412	p
**Age at pregnancy, years (median** **{IQR})**	29.00 {24.00, 33.00}	27.00 {21.00, 32.00}	<0.001
**Post pregnancy diabetes (%)**	4,169 (29.6)	16,741 (23.8)	<0.001
**Post pregnancy hypertension** **(%)**	5,592 (39.7)	9,347 (13.3)	<0.001
**Gestational diabetes (%)**	377 (2.7)	521 (0.7)	<0.001
**IMD Quintile (%)**			<0.001
**1**	2,698 (19.2)	11,015 (15.6)	
**2**	2,178 (15.5)	10,656 (15.1)	
**3**	2,971 (21.1)	14,671 (20.8)	
**4**	3,182 (22.6)	16,748 (23.8)	
**5**	3,054 (21.7)	17,322 (24.6)	
**Parity (categorical) (%)**			<0.001
**1**	3,822 (27.1)	26,401 (37.5)	
**2**	4,336 (30.8)	18,504 (26.3)	
**3**	2,759 (19.6)	11,341 (16.1)	
**>4**	3,166 (22.5)	14,166 (20.1)	
**Ethnicity (%)**			<0.001
**Not stated**	1,937 (13.8)	13,452 (19.3)	
**White**	9,321 (66.2)	42,486 (61.1)	
**Black**	1,143 (8.1)	4,235 (6.1)	
**South Asian**	1,140 (8.1)	5,946 (8.6)	
**Mixed**	258 (1.8)	1,349 (1.9)	
**Other**	284 (2.0)	2,071 (3.0)	

IMD= indices of multiple deprivation. Quintile 1 is the most deprived, and quintile 5 is the least deprived. HDP = hypertensive disorder of pregnancy.

## Key Points

### Question

What is the association between hypertensive disorders of pregnancy and the development of dilated cardiomyopathy?

### Findings

In this UK population-based cohort study, hypertensive disorders of pregnancy are associated with a twofold greater hazard of developing dilated cardiomyopathy. Dilated cardiomyopathy develops on average 5 years after the hypertensive disorder of pregnancy. Older maternal age and incident hypertension post-partum are associated with the development of dilated cardiomyopathy in at risk women.

### Meaning

Long term clinical vigilance of women with a history of hypertensive disorders of pregnancy for the development of dilated cardiomyopathy may be warranted.

## Abbreviations

CPRD: Clinical Practice Research Datalink
DCM: dilated cardiomyopathy
EHR: electronic health records
HES: hospital episode statistics
HDP: hypertensive disorders of pregnancy
