# Supplementary material for: Association Between Hypertensive Disorders of Pregnancy and Long-term Risk of Dilated Cardiomyopathy: Population based cohort study using multiple linked UK electronic health records databases
Source: JAMA Cardiol. Author manuscript; Available in PMC 2025 Apr 4. (PMC11966468; doi:10.1001/jamacardio.2025.0328)
Supplement: Supplementary [file EMS203140-supplement-Supplementary_.docx]

**Association Between Hypertensive Disorders of Pregnancy and Long-term Risk of Dilated Cardiomyopathy: Population based cohort study using multiple linked UK electronic health records databases**

**Supplementary Materials**

**Supplementary Methods p2 - 5**

**Supplementary Results p6**

**Supplementary Figures p7-8**

**Supplementary Tables p9-15**

**Supplementary References p16**

**Methods**

**Data sources: *CPRD primary care data, linked datasets and the Pregnancy Register***

We used routinely collected healthcare data from the Clinical Practice Research Datalink (CPRD) Aurum national primary care database,  containing deidentified patient information from consenting general practices (GPs) across the UK. As of 2023, the database included 45 million research acceptable patients. CPRD Aurum is broadly representative of the UK population in terms of age, sex and ethnicity^1-3^.  CPRD contains detailed data on patient demographics, diagnoses, prescription records, laboratory tests and referrals to specialist care. Primary care data from the July 2023 build were linked by a trusted third party via patient residential postcode to small-area-level data for socioeconomic deprivation measures^4^.

***IMD***

IMD is a composite measure derived from a number of ‘domains’ covering different aspects of material deprivation including health, employment, income, education and skills, housing, crime, living environment and access to services. The overall composite index is calculated as a weighted sum of the individual domain scores.

Linkage to secondary care data was available from NHS England’s Hospital Episode Statistics (HES) Admitted Patient Care (APC), and death registrations, including cause of death information, from the Office of National Statistics mortality database^5^. HES APC (Admitted Patient Care) data includes information on admission and discharge dates, diagnoses, specialists seen, and procedures undertaken.

The CPRD Aurum Pregnancy Register contains a list of all pregnancy episodes within the CPRD Aurum database^6, 7^. Developed using an algorithm, it contains data on the start and end of pregnancy, trimester dates and the outcome of the pregnancy. There may be more than one pregnancy episode per woman and approximately 17 million pregnancy episodes are recorded.

**Study Population**

The study population was derived from individuals in the Pregnancy Register who were contributing data to CPRD Aurum and HES linked data. We included all individuals with a HDP (pre-eclampsia and gestational hypertension) in a first pregnancy with a known outcome of live birth or stillbirth from the pregnancy register. An unexposed cohort of individuals was randomly sampled from the pregnancy register in a 5:1 ratio. These were individuals with a first pregnancy without HDP in their first or any pregnancy. The pregnancy time period was set from 1^st^ Jan 1997 to 31^st^ December 2018 to allow at least 5 years of follow up to 31^st^ July 2023 for the majority of the cohort (Supplementary Figure 2).

**Exposures**

HDP were defined using diagnostic code lists (eTable 1). Pre-eclampsia and gestational hypertension were considered together in the primary analysis, and pre-eclampsia and severe pre-eclampsia were considered separately in sensitivity analyses.

**Outcome**

Participants fulfilling the inclusion criteria had their electronic records searched for DCM developing after the end of the pregnancy. DCM was defined using clinical diagnostic ‘medcodes’ based on SNOMED-CT codes for primary care, and ICD10 codes for secondary care (eTable 1).To capture DCM that had been coded as heart failure, non-ischaemic heart failure was captured through the inclusion of heart failure codes and the exclusion of ischaemic heart disease and other secondary causes of heart failure (including valvular heart disease and hypertensive heart failure) (eTable 1).

For all analyses of each outcome, participants were followed up from the index date (end of pregnancy) until the minimum of the outcome of interest, leaving the practice, death or practice last collection date.

**Covariates**

The following covariates were derived using the Pregnancy Register, CPRD Aurum and HES datasets and included in analyses: maternal age at time of first pregnancy, birth year, gestational diabetes, post pregnancy diabetes, post pregnancy hypertension, total parity, socio-economic status and ethnicity.

Socioeconomic status for each participant was based on the 2019 English Index of Multiple Deprivations (IMD) linked to the patient’s postcode, grouped into quintiles (Supplementary methods). A score of one denotes ‘most deprived’ and five denotes ‘least deprived’. Participant ethnicity was defined from CPRD Aurum (and HES datasets if there was no valid code recorded in CPRD), as the most commonly recorded ethnicity value across all datasets.

Birth year, obtained from the Pregnancy Register, was used to account for variations in healthcare practice over time.

Clinical diagnostic codes (medcode) were used to define the presence of clinical comorbidities including gestational diabetes, post pregnancy diabetes, post pregnancy hypertension (eTable 1). The comorbidity was classified as being present (or absent) based on the presence (or not) of the relevant code at any point during follow-up.

**Validity of clinical diagnoses in CPRD**

The validity of clinical diagnoses recorded in CPRD has been investigated for a wide range of diseases, including heart failure^8^ and other common chronic conditions, with an average positive predictive value of 89% and 92% completeness, compared with national registry data^9, 10^.

Pregnancy-related hypertension is reported with a reasonable level of accuracy across population health datasets such as these^11^. HDP are identified with high specificity ( ≥98%); however, their sensitivity varies widely—especially for HDP subtypes (lower for gestational hypertension 10% than pre-eclampsia 75%)^12^, which is why the primary analysis in our study grouped HDP together.

Meta-analysis of heart failure studies showed that routinely collected healthcare data have high specificity (96.2%, 95% confidence interval [CI] 91.5–98.3) for heart failure events, and similar to HDP, less sensitivity (63.5%, 95% CI 51.3–74.1)^13^.

**Statistical analysis**

Baseline characteristics of the cohort were summarised. Mean (SD) or median (IQR) for continuous variables were used and compared using t-tests or Mann Whitney U tests as appropriate. Counts and percentages were used for categorical and binary variables and compared using Fisher’s or χ^2^ tests as appropriate.

To relate HDP history to risk of DCM outcome, we fitted Cox proportional hazards models with time between the end of pregnancy to cardiomyopathy outcome as duration. We report unadjusted hazard ratios, and subsequently adjusted for risk factors and demographics listed in Table 1. The proportional hazards assumption was tested on the basis of Schoenfeld residuals (using the *estat* test). Sensitivity analyses restricted to those with a history of pre-eclampsia only, excluding gestational hypertension.

All analyses were done using Stata, version 17.

**Ethics**

CPRD has NHS Health Research Authority (HRA) Research Ethics Committee (REC) approval to allow the collection and release of anonymized primary care data for observational research (NHS HRA REC reference number: 05/MRE04/87). Each year CPRD obtains Section 251 regulatory support through the HRA Confidentiality Advisory Group (CAG), to enable patient identifiers, without accompanying clinical data, to flow from CPRD contributing GP practices in England to NHS Digital, for the purposes of data linkage (CAG reference number: 21/CAG/0008). The protocol for this research was approved by CPRD’s Research Data Governance (RDG) Process (protocol number: 23_002823) and the approved protocol is available upon request. Linked pseudonymized data was provided for this study by CPRD. Data is linked by NHS Digital, the statutory trusted third party for linking data, using identifiable data held only by NHS Digital. Select general practices consent to this process at a practice level with individual patients having the right to opt-out.

**Data Availability**

Data are available on request from the CPRD. Their provision requires the purchase of a license, and this license does not permit the authors to make them publicly available to all. This work used data from the version collected in July 2023 (protocol #23_002823) and have clearly specified the data selected within each Methods section. To allow identical data to be obtained by others, via the purchase of a license, the code lists will be provided upon request. Licenses are available from the CPRD (<http://www.cprd.com>): The Clinical Practice Research Datalink Group, The Medicines and Healthcare products Regulatory Agency, 10 South Colonnade, Canary Wharf, London E14 4PU.

**Supplementary Results**

*Recurrent events*

We were not able to evaluate the risk of developing DCM with the cumulative number of pregnancies affected by HDP due to the limited number of individuals with an affected second pregnancy (n=341).

**Supplementary figure**

**SF1:**


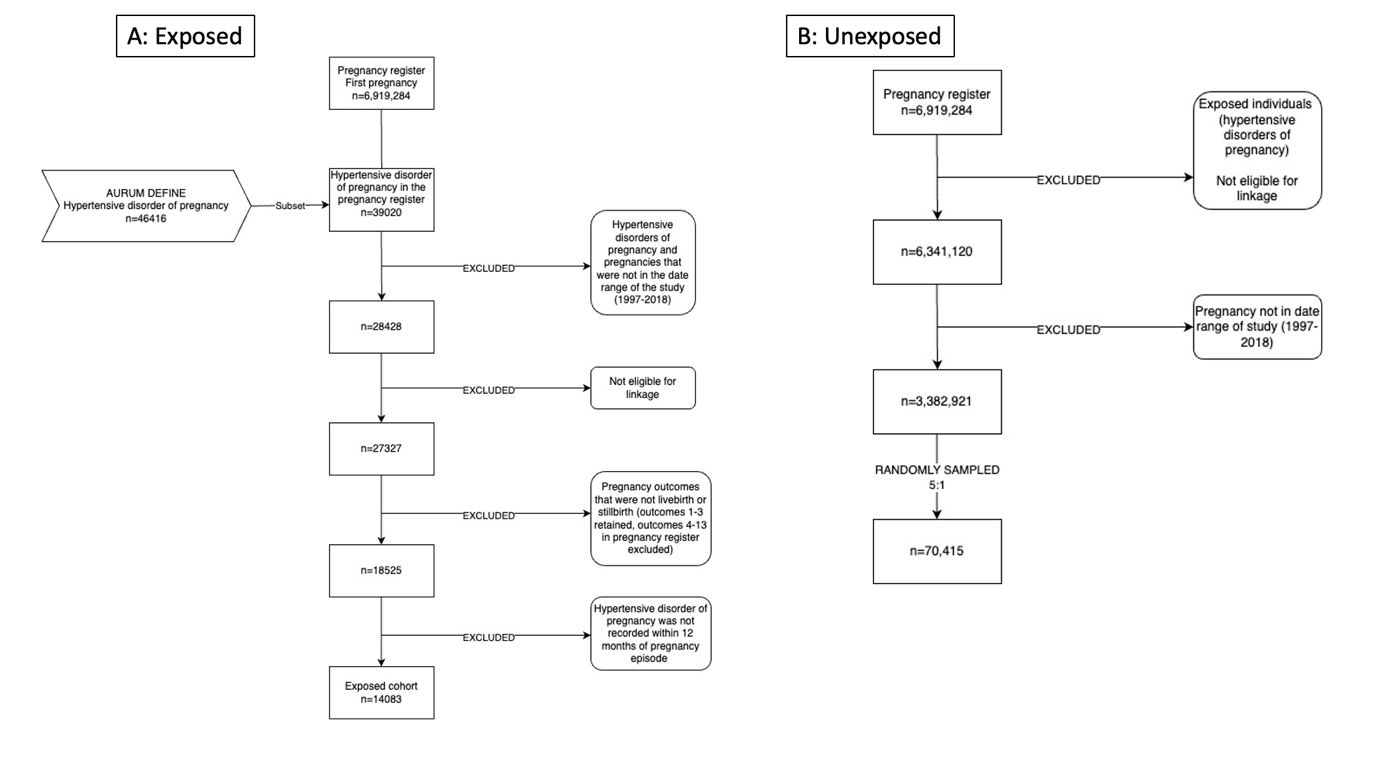


Flowchart outlining (Panel A) the assembly of the exposed cohort – women who experienced a hypertensive disorder of pregnancy during their first pregnancy and (Panel B) the unexposed cohort – women who did not experience a hypertensive disorder of pregnancy during their first pregnancy.

**Supplementary Tables**

eTable 1: Codelists and ICD10 codes (excel workbook)

**Supplementary Table 1:** Full Cox model of association between hypertensive disorder of pregnancy and development of dilated cardiomyopathy (DCM).

| **Variable** | | **Hazard ratio of developing DCM** | **95% CI** | **P value** |
| --- | --- | --- | --- | --- |
| **Hypertensive disorder of pregnancy** | | 1.55 | 1.04 -2.31 | 0.031 |
| **Maternal age at pregnancy (per year)** | | 1.06 | 1.03- 1.08 | <0.001 |
| **Year of birth (reference 1997)** | | 1.10 | 1.06- 1.15 | <0.001 |
| **Post pregnancy diabetes (present)** | | 1.03 | 0.70- 1.51 | 0.890 |
| **Post pregnancy hypertension (present)** | | 1.63 | 1.10- 2.42 | 0.015 |
| **Gestational diabetes (present)** | | 1.58 | 0.49- 5.10 | 0.441 |
| **Final parity** | | 1.01 | 0.92 -1.11 | 0.807 |
| **IMD quintile (reference 1)** | **2** | 2.06 | 1.07- 3.97 | 0.030 |
|  | **3** | 1.34 | 0.68- 2.64 | 0.397 |
|  | **4** | 2.11 | 1.13- 3.95 | 0.019 |
|  | **5** | 2.13 | 1.14 -4.00 | 0.018 |
| **Ethnicity**  **(reference ‘Not Stated’)** | **White** | 0.55 | 0.31 -0.97 | 0.038 |
|  | **Black** | 0.92 | 0.43 -1.94 | 0.810 |
|  | **South Asian** | 0.80 | 0.38 -1.66 | 0.549 |
|  | **Mixed** | 0.99 | 0.32 - 3.01 | 0.980 |
|  | **Other** | 0.36 | 0.08 - 1.58 | 0.174 |

**Supplementary Table 2:** Full Cox model of association between pre-eclampsia and development of dilated cardiomyopathy (DCM).

| **Variable** | | **Hazard ratio of developing DCM** | **95% CI** | **P value** |
| --- | --- | --- | --- | --- |
| **Pre-eclampsia** | | 1.85 | 1.24 - 2.76 | 0.002 |
| **Maternal age at pregnancy (per year)** | | 1.06 | 1.03 -1.08 | <0.001 |
| **Year of birth (reference 1997)** | | 1.10 | 1.06 -1.15 | <0.001 |
| **Post pregnancy diabetes (present)** | | 1.02 | 0.70 -1.50 | 0.916 |
| **Post pregnancy hypertension (present)** | | 1.68 | 1.15 -2.48 | 0.008 |
| **Gestational diabetes (present)** | | 1.64 | 0.51 - 5.27 | 0.404 |
| **Final parity** | | 1.01 | 0.92 -1.11 | 0.808 |
| **IMD quintile (reference 1)** | **2** | 2.07 | 1.07 -3.98 | 0.030 |
|  | **3** | 1.34 | 0.68 -2.65 | 0.394 |
|  | **4** | 2.14 | 1.14 -4.00 | 0.017 |
|  | **5** | 2.15 | 1.15 -4.04 | 0.017 |
| **Ethnicity**  **(reference ‘Not Stated’)** | **White** | 0.55 | 0.31 - 0.97 | 0.039 |
|  | **Black** | 0.92 | 0.43-1.95 | 0.823 |
|  | **South Asian** | 0.81 | 0.39-1.68 | 0.573 |
|  | **Mixed** | 0.99 | 0.32-3.02 | 0.983 |
|  | **Other** | 0.36 | 0.08 -1.60 | 0.181 |

**Supplementary Table 3:** Cox model of association between hypertensive disorders of pregnancy and development of (i) all cause heart failure and (ii) atherosclerotic cardiovascular disease.

|  |  | **All-cause Heart Failure** | | | **Atherosclerotic cardiovascular disease (ASCVD** | | |
| --- | --- | --- | --- | --- | --- | --- | --- |
| **Model** | **Variable** | Hazard ratio of developing all-cause heart failure | 95% CI | P value | Hazard ratio of developing ASCVD | 95% CI | P value |
| **Model 1: Hypertensive disorders of pregnancy only** | **Hypertensive disorders of pregnancy** | 2.03 | 1.43 to 2.89 | <0.0001 | 1.63 | 1.33 to 2.00 | <0.001 |
| **Model 2: Hypertensive disorders of pregnancy and maternal age** | **Hypertensive disorders of pregnancy** | 1.87 | 1.31 to 2.67 | 0.001 | 1.48 | 1.20 to 1.81 | <0.001 |
| **Model 3: Fully adjusted including maternal age, year of birth, post pregnancy diabetes, incident hypertension, gestational diabetes, parity, ethnicity, IMD quintile** | **Hypertensive disorders of pregnancy** | 1.48 | 1.01 to 2.15 | 0.042 | 1.22 | 0.98 to 1.51 | 0.07 |

**Supplementary Table 4: The effect of incident hypertension on the hazard of developing DCM.** With respect to persistent hypertension after delivery, on univariable analysis, incident hypertension was associated with the development of dilated cardiomyopathy. When including hypertensive disorders of pregnancy in the model, incident hypertension remains associated with the development of DCM, though the effect size is attenuated for both incident hypertension and hypertensive disorders of pregnancy. In the fully adjusted model, both hypertensive disorders of pregnancy and incident hypertension have a similar effect size for the hazard ratio of developing DCM.

| **Model** | **Variable** | **Hazard ratio of developing DCM** | **95% CI** | **P value** |
| --- | --- | --- | --- | --- |
| **Model 1: Incident hypertension only** | **Incident hypertension** | 1.92 | 1.35 to 2.73 | <0.0001 |
| **Model 2: Hypertensive disorders of pregnancy only** | **Hypertensive disorders of pregnancy** | 2.11 | 1.45 to 3.06 | 0.0002 |
| **Model 3: Incident hypertension and hypertensive disorders of pregnancy** | **Incident hypertension** | 1.68 | 1.16 to 2.42 | 0.006 |
|  | **Hypertensive disorders of pregnancy** | 1.83 | 1.24 to 2.69 | 0.002 |
| **Model 4: Fully adjusted including maternal age, year of birth, post pregnancy diabetes, incident hypertension, gestational diabetes, parity, ethnicity, IMD quintile** | **Hypertensive disorders of pregnancy** | 1.55 | 1.04 -2.31 | 0.031 |
|  | **Post pregnancy hypertension (present)** | 1.63 | 1.10- 2.42 | 0.015 |

**Supplementary References**

1. *CPRD Aurum 2024: Primary care data for public health research*. <https://www.cprd.com/primary-care-data-public-health-research>.

2. Wolf A, Dedman D, Campbell J, Booth H, Lunn D, Chapman J, Myles P. Data resource profile: Clinical Practice Research Datalink (CPRD) Aurum. International Journal of Epidemiology 2019;**48**(6):1740-1740g.

3. CPRD Aurum July 2023 build Clinical Practice Research Datalink 2023.

4. Mahadevan P, Harley M, Fordyce S, Hodgson S, Ghosh R, Myles P, Booth H, Axson E. Completeness and representativeness of small area socioeconomic data linked with the UK Clinical Practice Research Datalink (CPRD). Journal of Epidemiology and Community Health 2022;**76**(10):880-886.

5. Padmanabhan S, Carty L, Cameron E, Ghosh RE, Williams R, Strongman H. Approach to record linkage of primary care data from Clinical Practice Research Datalink to other health-related patient data: overview and implications. European Journal of Epidemiology 2019;**34**(1):91-99.

6. Minassian C, Williams R, Meeraus WH, Smeeth L, Campbell OMR, Thomas SL. Methods to generate and validate a Pregnancy Register in the UK Clinical Practice Research Datalink primary care database. Pharmacoepidemiol Drug Saf 2019;**28**(7):923-933.

7. Campbell J, Shepherd H, Welburn S, Barnett R, Oyinlola J, Oues N, Williams R. Methods to refine and extend a Pregnancy Register in the UK Clinical Practice Research Datalink primary care databases. Pharmacoepidemiol Drug Saf 2023;**32**(6):617-624.

8. Johansson S, Wallander MA, Ruigomez A, Garcia Rodriguez LA. Incidence of newly diagnosed heart failure in UK general practice. Eur J Heart Fail 2001;**3**(2):225-231.

9. Conrad N, Judge A, Tran J, Mohseni H, Hedgecott D, Crespillo AP, Allison M, Hemingway H, Cleland JG, McMurray JJV, Rahimi K. Temporal trends and patterns in heart failure incidence: a population-based study of 4 million individuals. The Lancet 2018;**391**(10120):572-580.

10. Herrett E, Thomas SL, Schoonen WM, Smeeth L, Hall AJ. Validation and validity of diagnoses in the General Practice Research Database: a systematic review. Br J Clin Pharmacol 2010;**69**(1):4-14.

11. Roberts CL, Bell JC, Ford JB, Hadfield RM, Algert CS, Morris JM. The accuracy of reporting of the hypertensive disorders of pregnancy in population health data. Hypertens Pregnancy 2008;**27**(3):285-297.

12. Amy J, Sonia RD, Victrine T, Becky S, Peter T, Graeme NS, Thais C, Jodi DE. Systematic review of validated case definitions to identify hypertensive disorders of pregnancy in administrative healthcare databases. Open Heart 2023;**10**(2):e002151.

13. Goonasekera MA, Offer A, Karsan W, El-Nayir M, Mallorie AE, Parish S, Haynes RJ, Mafham MM. Accuracy of heart failure ascertainment using routinely collected healthcare data: a systematic review and meta-analysis. Systematic Reviews 2024;**13**(1):79.
